# Supplementary material for: Functional, Antioxidant, and Anti-Inflammatory Properties of Cricket Protein Concentrate (Gryllus assimilis)
Source: Biology (Basel). 2022 May 20;11(5):776. doi: 10.3390/biology11050776 (PMC9138711; doi:10.3390/biology11050776)
Supplement: Supplementary file 1 [file biology-11-00776-s001.zip › biology-1707029-SI.pdf]

Cricket protein isolate (CPI) blue Coomassie CPI photo of gel analyzer

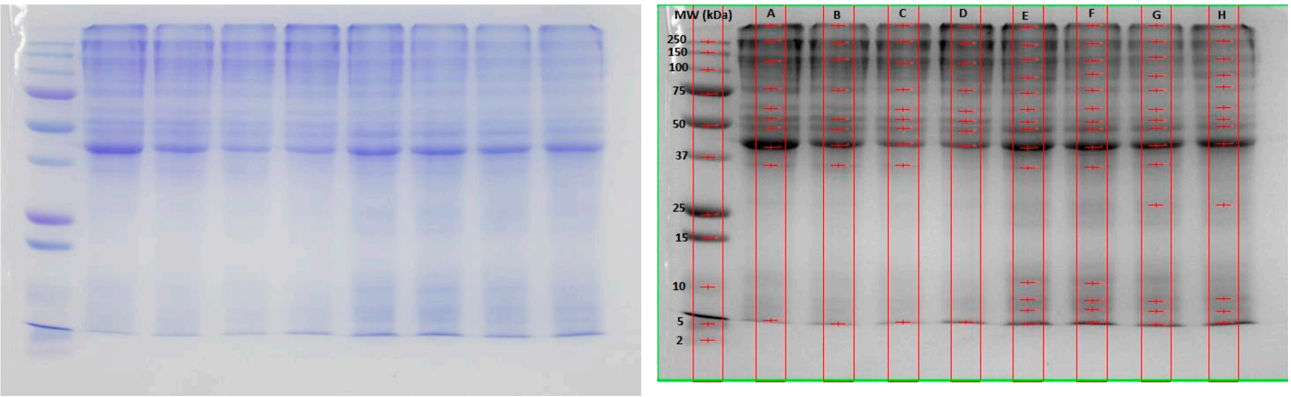

instrument

Figure S1. Cricket protein concentrate (CPC), the uncropped western blot figures were presented.

Gastric hydrolysate

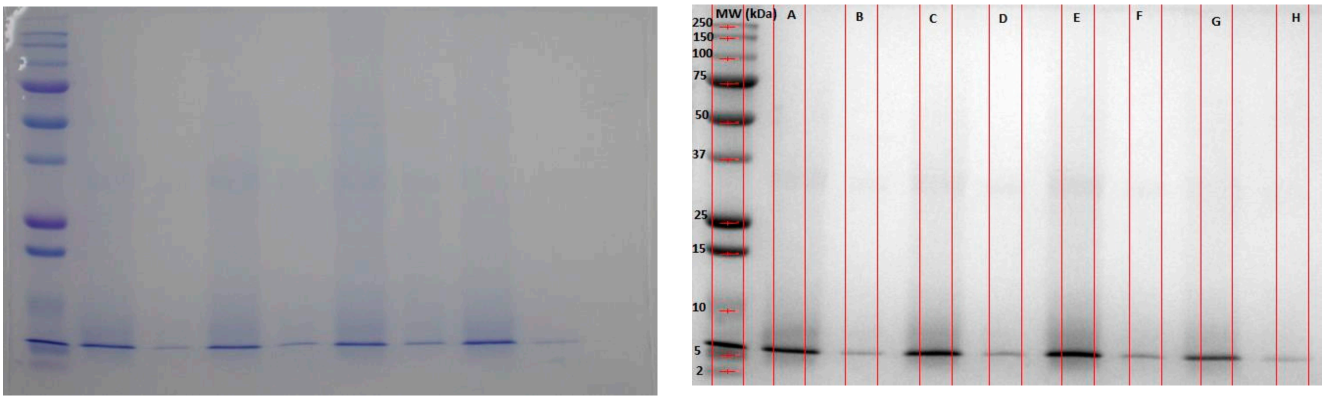

Duodenal hydrolysate

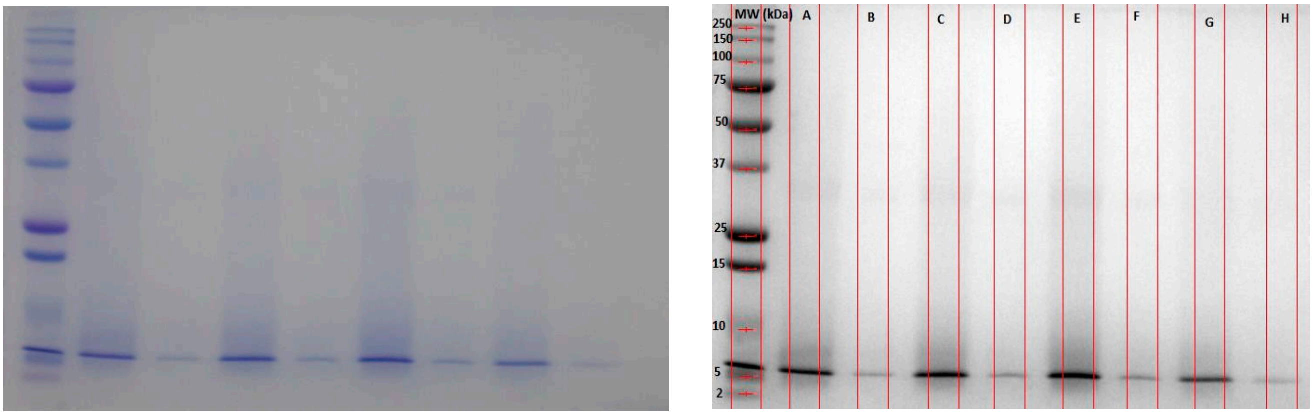

Figure S2. hydrolysates from cricket protein concentrate (CPC).
